# Increased risk of respiratory events during endobronchial ultrasound examination in patients with reduced forced expiratory volume: a prospective observational study

**DOI:** 10.3389/fmed.2024.1409160

**Published:** 2024-08-14

**Authors:** Achim Grünewaldt, Gernot Rohde

**Affiliations:** Department of Respiratory Medicine and Allergology, University Hospital, Goethe University, Frankfurt, Germany

**Keywords:** endobronchial ultrasound, monitoring, sedation, hypercapnia, desaturation

## Abstract

**Background:**

The incidence of adverse events during endobronchial ultrasound is low. Nevertheless, it is unclear, whether patients with impaired pulmonary function have an increased risk of respiratory events during the intervention.

**Methods:**

A monocentric prospective observational study was performed at the Department of Respiratory Medicine, University Hospital Frankfurt/Main, Germany. Adult patients undergoing an endobronchial ultrasound examination with propofol-sedation were included. Pre-interventional screening included pulmonary function testing, laboratory tests and electrocardiogram. The occurrence of hypercapnia >55 mmHg or reduced oxygen saturation <85% was defined as a respiratory event was recorded and compared between patients with normal and impaired pulmonary function tests.

**Results:**

In total, 126 patients were included. Pulmonary function testing revealed a median FEV1 of 2.2 l (range 0.4–6.04l) and a predicted FEV1 of 79.5% (range 20–127.8%) respectively. The median FVC was 3.0 l (range 0.87–7.28l), the median predicted FVC was 82% (range 31.4–128.4%). In 72 examinations (60%) pCO_2_ levels >55 mmHg were measured. Transient oxygen desaturation <85% occurred in 31 cases (25.8%). The Mann Whitney U-test showed a significantly lower FEV1 (% predicted value) in patients with respiratory events (*p* = 0.007). ROC analysis identified a predicted FEV1 of 78.5% as the optimal cut-off with a sensitivity of 58% and a specificity of 71%. Using Z-score instead of predicted values, there was no significant association between a lower Z- score of FEV or FVC and hypercapnic or hypoxic events. However, both a lower absolute value of FEV1/FVC and a lower Z-score of the FEV1/FVC index were associated with the occurrence of respiratory events. In binary logistic regression analysis, we could not demonstrate any association with other relevant parameters (age, BMI, sedation dosage, sedation duration, or ASA-score).

**Conclusions:**

An impaired forced expiratory volume is associated with the frequency of respiratory events during endobronchial ultrasound examination under propofol-sedation.

## Introduction

Endobronchial ultrasound examination is an established diagnostic standard for the sampling of histological specimens from peribronchial and mediastinal structures (1). This is particularly true for endosonographic staging of lymph nodes in malignant thoracic diseases (2). In general, complication rates of bronchoscopic interventions including endobronchial ultrasound are rare and primarily concern infections, bleeding or pneumothorax (3). However, results from several studies indicate that a decrease in oxygen saturation is a frequent event during bronchoscopic examinations (4, 5).

There is no national or international standard operating procedure for this examination (6). Many respiratory departments perform the examination using propofol-sedation and endotracheal intubation with a specific endotracheal tube, such as the Bronchoflex^®^ tube (Rüsch^®^). A more recent study by Mercado et al. showed a comparable safety profile of non-anaesthesiologist-administered propofol to midazolam-sedation for bronchoscopic interventions (7).

A pre-interventional pulmonary function test, routine laboratory tests including kidney function parameters and an electrocardiogram are usually required.

Risk factors such as older age, active cigarette smoking or the presence of chronic comorbidities such as chronic obstructive pulmonary disease (COPD), or cardiovascular disease are common in patients presenting for bronchoscopic examination.

In particular, COPD is a common underlying disease in patients undergoing endobronchial ultrasound. Many patients receive the procedure following suspicious nodules on chest computed tomography. Due to the common underlying risk factor of smoking, the comorbidity of COPD and lung cancer is high (8). COPD patients with severe pulmonary function impairment are at increased risk of developing respiratory failure, including progression of impaired respiratory muscle function and subsequent hypercapnia. Therefore, although the current COPD assessment according to the Global Initiative for Chronic Obstructive Lung Disease (GOLD) focuses more on the occurrence of exacerbations and the patient's symptoms, the impairment of pulmonary function testing is still taken into account with GOLD-stages I-IV (9). It can be assumed that especially patients with severe reduction of expiratory flow have an increased risk of respiratory failure and therefore may experience more respiratory events during bronchoscopic procedures.

Nevertheless, to our knowledge, the possible impact of impaired pulmonary function on the occurrence and frequency of respiratory events or other adverse events has not been studied yet. Understanding this better holds the potential to identify risk profiles and to improve the examination modalities i.e., by employing extended monitoring or anesthesiologic support. There are several established methods for monitoring respiratory parameters during endoscopic procedures. While the measurement of peripheral oxygen saturation is standard of care in most endoscopic units, the measurement of pCO_2_ varies. Measurement of end-tidal CO_2_ is usually performed in patients undergoing endoscopic procedures under general anesthesia. At the study center, endobronchial ultrasound is performed in most cases under sedation and spontaneous ventilation. The endobronchial tube is blocked by the endoscope for a relevant part of the examination time. This can interfere with the end tidal measurement of pCO_2_. Therefore, we use transcutaneous measurement of pCO_2_ which reflects reliable pCO_2_-levels and has been evaluated in endoscopic procedures (10, 11). Several studies have shown that there is a good correlation between arterial paCO_2_-measurement and transcutaneous pCO_2_-detection (12, 13).

The aim of this prospective observational study was to assess the parameters that delineate the peri-interventional risk profile and to relate this to the occurrence of respiratory events during the endobronchial ultrasound examination (defined as hypercapnia or decrease of oxygen saturation).

## Material and methods

All candidates gave written informed consent, and the study protocol was approved by the local ethics committee (study number 148-16).

### Study design and subjects

This monocentric prospective observational study was conducted at the Department of Respiratory Medicine of the University Hospital of the Goethe University Frankfurt/Main.

During the period from 05/2016 until 08/2020 we consecutively recruited adult patients who underwent an endobronchial ultrasound examination. Exclusion criteria were pCO_2_ > 55 mmHg, SpO_2_ <85%, and systolic blood pressure <80 mmHg before the start of the examination. Furthermore, patients on invasive mechanical ventilation and receiving muscle relaxants were excluded.

Pre-interventional screening procedures included pulmonary function testing, ECG and routine blood tests including creatinine-level, blood count and coagulation-testing.

### Intervention

In all procedures diagnostic ultrasound was performed first. If appropriate, histologic specimens were obtained by transbronchial needle aspiration (TBNA, Olympus 19 or 21G needle).

Patients underwent continuous non-invasive monitoring including non-invasive blood pressure measurement at regular 3 min-intervals, ECG monitoring, and peripheral pulse oximetry (GE Healthcare Patient Monitoring). pCO_2_ was measured continuously non-invasively by transcutaneous pCO_2_-monitoring (Sentec). According to the study protocol, respiratory events were defined as an increase of pCO2 > 55 mmHg or a decrease in SpO2 level <85%. These events were documented on a paper case report form by the physician administering the sedation therapy. Patients with SpO_2_- levels <85% or pCO_2_-levels > 55 mmHg prior to sedation were excluded from the study.

The endoscopic procedure was performed under sedation with midazolam and propofol. Sedation was administered and monitored by a second physician. According to the sedation protocol, all patients received an initial dose of 3 mg midazolam and 1 mg/kg body weight propofol (maximum 50 mg). Sedation was maintained with propofol-boluses of 20 mg. Bolus application was a physician decision based on patient alertness (goal of sedation was feasibility of the bronchoscopic procedure). The minimum interval between the propofol boluses was 20 seconds. No maximum of propofol dose was defined for the study. Before intubation, preoxygenation was performed using an oxygen reservoir mask with an inspiratory oxygen fraction of nearly 100%. After intubation, oxygen was supplemented through an integrated tube channel. Flowrate was determined by the clinicians based on the measured peripheral oxygen saturation.

The patients were intubated with a Bronchoflex©-tube (Ruesch, Germany). Following the endotracheal intubation, local anesthesia using 10–20 ml lidocaine 0.5% was applied into the bronchial system.

Further variables assessed were anamnestic information about smoking-history, body weight, height, ASA-score, creatinine, duration of intervention, total amount of sedative applied, and need for transfer to intensive care unit.

### Data processing and statistical analysis

A paper case report form was used to document monitoring- and examination data. Additional information (pulmonary function results, laboratory results) was obtained by querying the electronic health records of the hospital data system “AGFA-Orbis.” Following the current recommendation of the ERS on interpretation standards for pulmonary function tests, we retrospectively calculated the Z-score of these test results in our cohort (https://gli-calculator.ersnet.org/docs.html) (14).

Data were electronically collected using Microsoft-Excel (Version Microsoft Home and Business 2016) and were transferred to SPSS (IBM SPSS Statistics version 27) for statistical analysis. The Kolmogorov-Smirnov-test was used to test for normal distribution. Continuous data are presented as median and range (minimum and maximum) and categorical data as percentages.

According to the study protocol, 125 patients were intended to enroll.

The hypothesis that patients with impaired pulmonary function results are at a higher risk to develop respiratory events compared to patients with normal pulmonary function test was tested by using Wilcoxon-Mann-Whitney-test. A *p*-value of <0.05 was considered significant. Further associations between the risk of hypercapnia during the intervention and clinical characteristics were examined by using logistic regression analysis.

### Patient and public involvement

Patients were not involved in the design, conduct, reporting, or dissemination plans of our research.

## Results

A total of 126 patients were included, 6 cases had to be rolled out retrospectively. The median age was 60 years (range 25–84 years), 77 patients (64.2%) were male. The mean BMI was 24.98 (±17.34). Forty six of 120 patients (38.3%) were active and 32 (26.6%) former smokers. The median creatinine level at admission was 0.86 mg/dl (range 0.33–5.65 mg/dl). The median absolute FEV1 was 2.2 l (range 0.4–6.04 l), the median predicted FEV1 % was 79.5% (range 20–127.8%). The median absolute FVC was 3.0 l (range 0.87–7.28 l), the median FVC % predicted was 82% (range 31.4–128.4%).

Table 1 summarizes the patients' sociodemographic and clinical characteristics.

**Table 1:** Patients characteristics including sociodemographic data and lung function results.

**Characteristics**		***p*-value**
**Demographics**		
Age [years], median (range)	60 (25–84)	0.87
Sex (male) *n* (%)	77 (64.2)	
Height [cm], mean (sd)	172.7 (9.85)	0.5624
Weight [kg], mean (sd)	77.2 (17.34)	0.454
BMI mean (sd)	24.98 (5.52)	0.224
Active smoker *n* (%)	46 (38.3)	0.054
Former smoker *n* (%)	32 (26.6)	0.239
**Laboratory results**		
Serum creatinine [mg/dl] median (range)	0.86 (0.33–5.65)	0.224
**Lungfunction results**		
FEV1 [l], median (range)	2.2 (0.4–6.04)	0.563
FEV1 (% predicted), median (range)	79.5 (20–127.8)	0.262
FEV1 z-score, median (range)	−1.07 (−4.8–3.65)	0.252
FEV1/FVC median (range)	73.33 (32.59–95.02)	0.774
FEV1/FVC z-score	−0.24 (−4.01–4.44)	0.911
FVC [l] median (range)	3.0 (0.87–7.28)	0.554
FVC (% predicted) median (range)	82 (31.4–128.4)	0.91
FVC z-score	−0.88 (−5.0–2.77)	0.506
**Degree of expiratory flow limitation**		0.413
FEV1 (% predicted) >80% *n* (%)	59 (49.2)	
FEV1 (% predicted) 50%−79% *n* (%)	38 (31.7)	
FEV1 (% predicted) 30%−49% *n* (%)	14 (11.7)	
FEV1 (% predicted) <30% *n* (%)	6 (5.0)	

sd, standard deviation; BMI, body mass index; FEV1, forced expiratory volume; FVC, forced vital capacity; *p*-value of logistic regression analysis with respiratory event as dependent variable.

Regarding the ASA status, 31 patients (25.8%) were assessed as ASA 1, 74 patients (61.7%) ASA 2, and 13 patients (10.8%) ASA 3.

In 72 examinations (60%) hypercapnic levels >55 mmHg were measured. In 31 cases (25.8%) transient oxygen desaturation <85% occurred. In all cases, hypercapnia and hypoxemia were temporarily and normalized after the examination. A post interventional ventilator therapy was not necessary in any case.

The mean propofol dosage was 407.5 mg (±177.8). The standardized midazolam dosage was 3 mg (in 9 cases higher levels of 5 mg were applied). The median examination time was 50 min (range 20–100 min). Due to a peri-interventional pneumothorax, in one case, a post-interventional ICU-transfer was necessary. Table 2 shows the results of the peri-interventional monitoring and examination characteristics.

**Table 2:** Results of peri-interventional monitoring.

**Characteristics**		***p*-value**
**ASA**		0.89
1	31 (25.8%)	
2	74 (61.7%)	
3	13 (10.8%)	
**Blood gas parameters**		
pCO2_2_ pre-interventional, median (range)	36.5 (27–51.9)	
Hypercapnia > 55 mmHg *n* (%)	72 (60)	
Oxygen-Desaturation during intervention *n* (%)	31 (25.8)	
Respiratory event (hypercapnia and/or desaturation)	82 (68.3%)	
**Endotracheal tube-diameter (mm)**		0.089
7.5 *n* (%)	23 (19.2)	
8.5 *n* (%)	94 (78.3)	
**Sedation**		
Midazolam [mg] median (range)	3 (3–5)^+^	
Propofol [mg] mean (sd)	407.5 (177.8)	0.996
Procedural duration [min] median (range)	50 (20–100)	0.624
Postinterventional ICU-admission *n* (%)	1 (0.8)^++^	

ASA, physical status system of the American society of anesthesiologists; ICU, intensive care unit; sd, standard deviation; ^+^in 9 cases 5 mg midazolam were applicated instead of the standardized dosage of 3 mg, *p*-value of logistic regression analysis; ^++^ICU-transfer due to pneumothorax.

The logistic regression analysis showed no significant association between patient characteristics, pulmonary function results or examination characteristics and the occurrence of hypercapnia or oxygen desaturation.

However, the Mann Whitney U-test revealed a significantly lower predicted FEV1 % in patients with hypercapnic events (*p*-value 0.012) and all respiratory events (hypercapnia and desaturation, *p*-value 0.007). The Mann Whitney U-test was not significant using the FEV1 Z-score as the test variable (*p*-value for hypercapnia 0.131 and for all respiratory events 0.151).

The FEV1/FVC index was significantly associated with respiratory events using the absolute value (*p* = 0.003) and the FEV1/FVC Z-score (*p* = 0.001).

**Figure 1:**
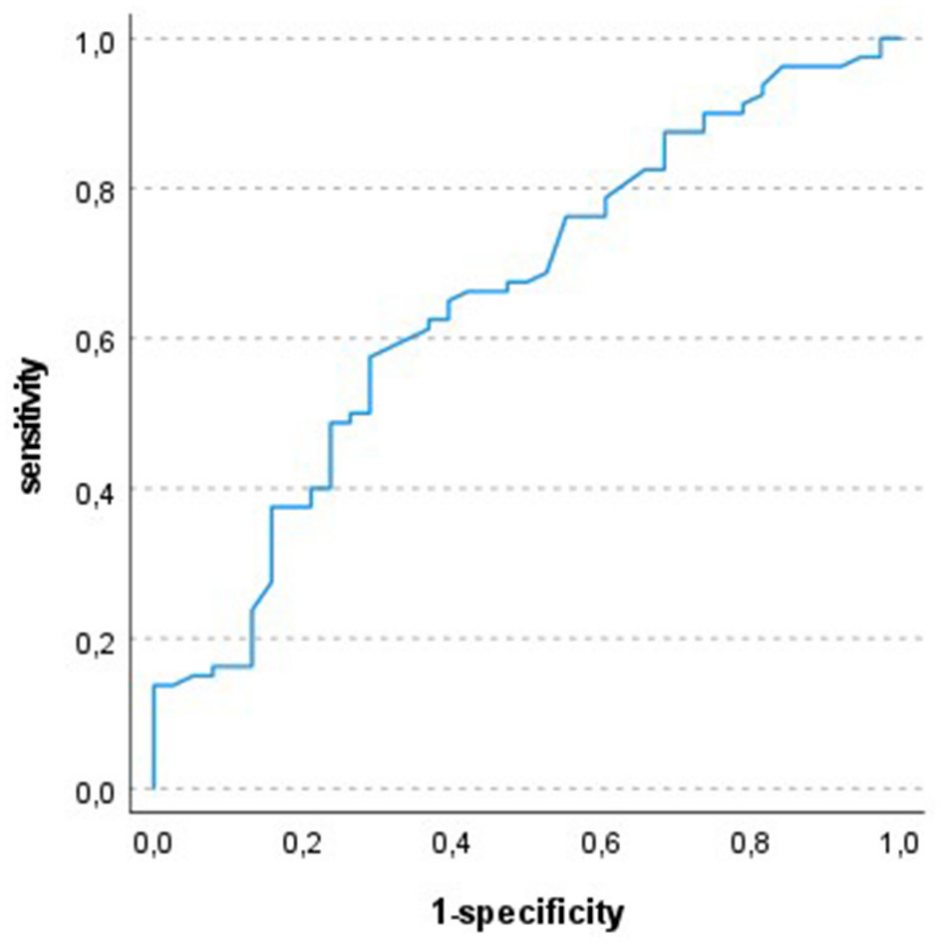
ROC-analysis: test variable FEV1 (% predicted), state variable respiratory event; optimal cut off 78.5%; AUC 0.653.

In ROC-analysis a FEV1 (%predicted) of 78.5% was identified as the optimal cut off with a sensitivity of 58% and a specificity of 71%. Figure 1 shows the ROC curve.

**Figure 2:**
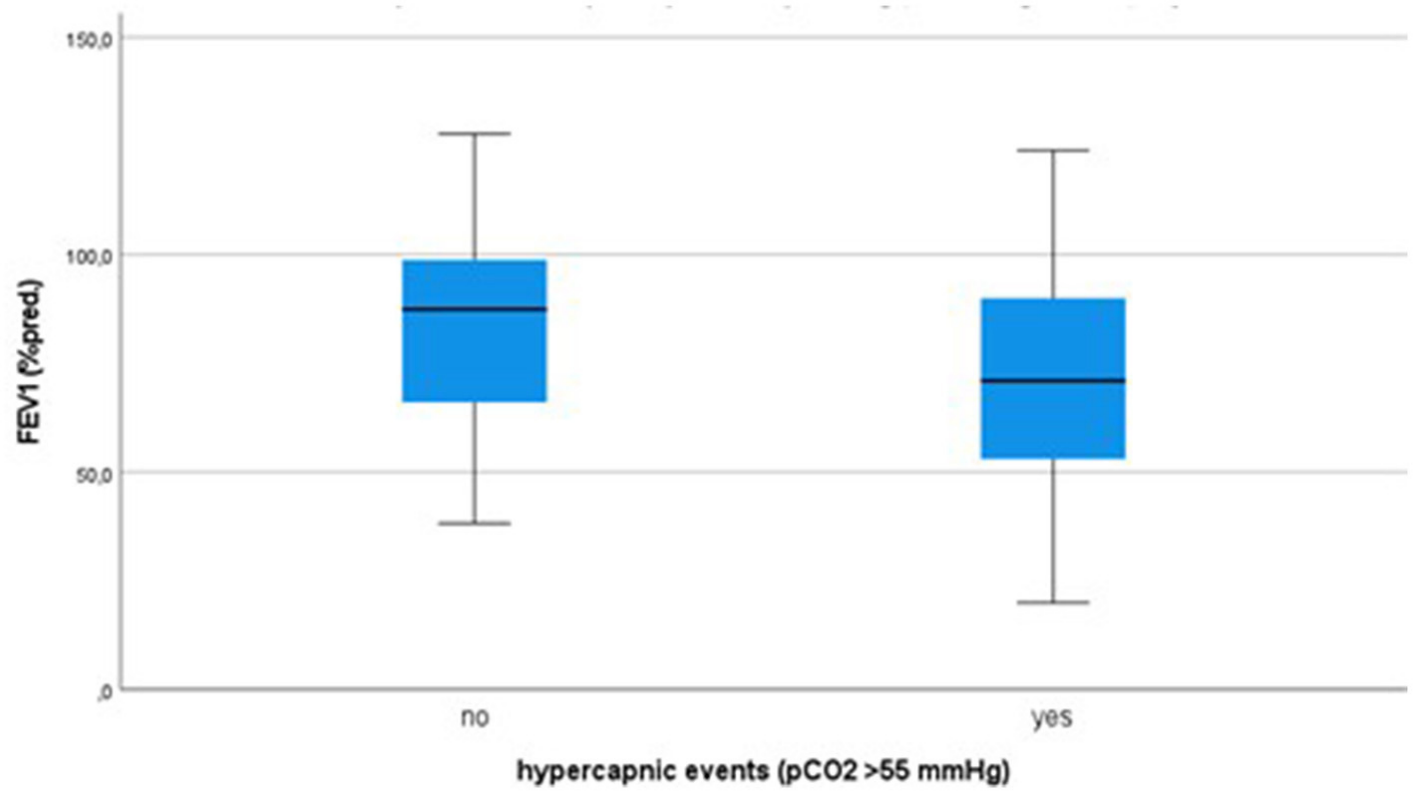
Distribution of FEV1 (% predicted value) in case of no occurrence of hypercapnic events and hypercapnic events > 55 mmHg, boxplots with median, 25/75%-quartiles and range.

The boxplots in Figure 2 illustrate the distribution of FEV1-values (% predicted) with and without the occurrence of hypercapnic events.

Furthermore, the association between the events “hypercapnia” and “hypoxemia” was examined with chi-square test. The results could not show correlation between both events (*p-*value 0.307). Table 3 gives the distribution of hypercapnia and hypoxemic events.

**Table 3:** Occurrence of hypercapnia and hypoxemic events.

		**Oxygen-desaturation**<**85%**		
		**No (n)**	**Yes (n)**	**Total** ***n*** **(%)**
Hypercapnia <55 mmHg	No (n)	38	10	48 (40%)
	Yes (n)	51	21	72 (60%)
	Total *n* (%)	89 (74.2%)	31 (25.8%)	120

Pearson-Chi-Quadrat = 0.307.

## Discussion

The study revealed the development of transient hypercapnia and hypoxemia as a common event during flexible bronchoscopy with endobronchial ultrasound, performed under intravenous sedation.

Chhajed et al. (11) published data about the development of hypoxemia and hypercapnia during flexible bronchoscopy. The sociodemographic distribution was similar to our cohort. Regarding the procedure (flexible bronchoscopy without endobronchial ultrasound) the examination time was much shorter. Hence, increasing pCO_2_ levels were observed in all cases (11).

More recent data from Galetin et al. demonstrated a relevant increase in pCO_2_ during endobronchial ultrasound in COPD patients (15). In accordance with our data, all patients recovered spontaneously from hypercapnia without the need for mechanical ventilation. In our cohort a lower FEV1/FVC index which reflects the presence of obstructive disturbance of ventilation, was associated with more frequent respiratory events, and, we demonstrated a rather weak association between expiratory flow limitation and the risk of respiratory events. The ROC-cut off with a predicted FEV1 of 78.5% demonstrates, that even a distinct limitation in pulmonary function results principally increase the risk of respiratory events.

In line with our data, Chhajed et al. (11) could not show any association between pCO_2_ levels and spO_2_ changes and COPD status. However, it should be considered that in contrast to the mentioned data, our focus was on the occurrence of hypercapnic or hypoxemic events and not on the absolute value of the pCO_2_-level and oxygen saturation.

Hence, in accordance to further studies, we could not demonstrate any association between sociodemographic parameters (age, sex) or patient constitution and the development of hypercapnia or hypoxemia (11).

In addition, we could not prove any association between pCO_2_-levels or hypoxemic episodes and dosage of sedation or duration of intervention. Similarly, Carmi et al. evaluated pCO_2_-changes between midazolam and propofol sedation and demonstrated a lower increase in carbon dioxide in the propofol group. Despite the difference in procedure (bronchoscopy vs. endobronchial ultrasound), this suggests that there may be less influence of propofol on ventilatory drive (16).

### What are the implications of our results for the clinical management of endobronchial ultrasound?

The results of our study suggest that any form of airflow limitation increases the risk of hypercapnia during flexible bronchoscopy in combination with endobronchial ultrasound. Hence, there is a weak association with an increased risk in patients with impaired pulmonary function. This underscores the importance of an accurate pre-interventional assessment of potential risk factors for peri-interventional complications. Our results confirm the feasibility of continuous transcutaneous CO_2_ monitoring as reported in former studies (11, 17). Especially in COPD patients, the peri-interventional transcutaneous pCO_2_-measurement should be standard of care. In conclusion, we can postulate that the pre-interventional pulmonary function test may have little impact on an increased risk of adverse events during bronchoscopy.

### What can we learn about the pathophysiological effects of pCO_2_- and spO_2_-abnormalities?

However, despite the development of hypercapnia, in no case postinterventional ventilatory assist was necessary. This illustrates that there is still a great lack of understanding of the effects of hypercapnia. While the development of acute hypercapnic failure (for example in COPD-exacerbation) is a strong indication for non-invasive ventilatory support, our cohort did not show any negative effects of this acute increase of hypercapnia. On account of recent data obtained by Chung et al. we suggest that several further factors contribute to the negative outcome in patients with acute respiratory acidosis (18).

In particular, the lack of association between the occurrence of hypercapnia and the decrease in oxygen saturation in our data underscores the completely different pathways of respiratory failure that could not be predicted by conventional lung function tests.

In our study we focused on the association between pulmonary function results and respiratory events. It may be clinically relevant if comorbidities such as heart failure or arterial hypertension have influence on sedation response and consequently respiratory events. Further studies evaluating the risk profile of endoscopic procedures should address this aspect in more detail.

## Conclusion

Our data revealed an increased incidence of hypercapnia in patients with reduced forced expiratory volume. As a consequence of our findings, we recommend peri-interventional transcutaneous CO_2_- monitoring as standard of observation, especially in COPD patients.

Recent data from a metanalysis by Sampsonas et al. suggest that high flow oxygen therapy reduces the risk of desoxygenation during bronchoscopic procedures (19). With regard to our results, these data may suggest further studies focusing on the effect of high flow oxygen therapy on the development of hypercapnia.

## Limitations

The study is subject to the inherent limitations of any monocentric-based data. Another omission is that there was no arterial blood gas analysis available to verify the measurements of the CO_2_- and SpO_2_- monitor analysis, and we did not evaluate the absolute values of the evolution of SpO_2_ and pCO_2_, but only our absolute cut-offs that we defined in our study protocol. Accordingly, other important blood gas parameters such as bicarbonate and pH are not included. These results could have supported the assessment of the development of ventilatory insufficiency with increasing levels of pCO_2_. Another missing was, that there was no standard procedure in case of the occurrence of a hypercapnic or hypoxemic event.

## Data availability statement

The raw data supporting the conclusions of this article will be made available by the authors on reasonable request.

## Ethics statement

The studies involving humans were approved by the Local Ethics Committee, University Hospital Frankfurt. The studies were conducted in accordance with the local legislation and institutional requirements. Written informed consent for participation was not required from the participants or the participants' legal guardians/next of kin in accordance with the national legislation and institutional requirements.

## Author contributions

AG: Writing – original draft, Conceptualization. GR: Writing – review & editing.
